# Effects of Intensive Impairment-Oriented Arm Rehabilitation for Chronic Stroke Survivors: An Observational Cohort Study

**DOI:** 10.3390/jcm14010176

**Published:** 2024-12-31

**Authors:** Thomas Platz, Katharina Kaiser, Tina Laborn, Michael Laborn

**Affiliations:** 1 Neurorehabilitation Research Group, University Medical Centre, 17475 Greifswald, Germany; 2 BDH-Klinik Greifswald, Institute for Neurorehabilitation and Evidence-Based Practice, “An-Institut”, University of Greifswald, 17491 Greifswald, Germany; 3 Hand and Occupational Therapy Outpatient Service Laborn, 80802 München, Germany

**Keywords:** rehabilitation, training, arm, stroke, clinical trial

## Abstract

**Objective**: To assess the effects of a two-week course of intensive impairment-oriented arm rehabilitation for chronic stroke survivors on motor function. **Methods**: An observational cohort study that enrolled chronic stroke survivors (≥6 months after stroke) with mild to severe arm paresis, who received a two-week course of impairment-oriented and technology-supported arm rehabilitation (1:1 participant–therapist setting), which was carried out daily (five days a week) for four hours. The outcome measures were as follows: the primary outcome was the arm motor function of the affected arm (mild paresis: BBT, NHPT; severe paresis: Fugl-Meyer arm motor score). The secondary outcomes were measures of finger strength, active ROM, spasticity, joint mobility/pain, somatosensation, emotional distress, quality of life, acceptability, and adverse events. **Results**: One hundred chronic stroke survivors (≥6 months after stroke) with mild to severe arm paresis were recruited. The training was acceptable (drop-out rate 3%; 3/100). The clinical assessment indicated improved motor function (SMD 0.42, 95% CI 0.36–0.49; n = 97), reduced spasticity/resistance to passive movement, and slightly improved joint mobility/pain and somatosensation. The technology-based objective measures corroborated the improved active range of motion for arm and finger joints, reduced finger spasticity/resistance to passive movement, and the increased amount of use in daily life, but there was no effect on finger strength. The patient’s emotional well-being and quality of life were positively influenced. Adverse events were reported by the majority of participants (51%, 49/97) and were mild. **Conclusions**: Structured intensive impairment-oriented and technology-supported arm rehabilitation can promote motor function among chronic stroke survivors with mild to severe arm paresis and is an acceptable and tolerable form of treatment when supervised and adjusted by therapists.

## 1. Introduction

Stroke is the third leading cause of death and disability, combined, in the world, and the burden it places on the healthcare system has increased substantially over the last few decades [1]. As a major cause of chronic impaired arm function, it frequently affects many activities of daily living. Between forty to seventy percent of those affected by stroke suffer from arm paresis initially [2,3]. Among those, two thirds have severe arm paresis [3]. Six months after stroke, the affected arm of approximately half of all stroke survivors, who initially had severe arm paresis, still remains without function [4]. Different training- and technology-based interventions have been shown to improve arm function after stroke [5,6] and are recommended for stroke rehabilitation [7]. Most spontaneous recovery and the best course of treatment in terms of improvements can be expected early after stroke, i.e., within the first three months, and when arm paresis is not severe [8,9]. And, while there is the potential for stroke survivors in the chronic phase to improve their motor function [10], it remains controversial how improvements to arm motor function can still be gained through training and whether improvements at this stage are related to the recovery of function, the enhancement of compensatory strategies, or a reversal of learnt non-use (only) [11].

This study followed the rationale (and hypothesis) that motor recovery, i.e., the improvement of motor control, such as selective movement control (rather than improved function due to compensatory behaviour), is still achievable by stroke survivors in the chronic stage when therapy offers training that explicitly, specifically, intensively, and comprehensively addresses the motor control to be regained, i.e., the ability to move the arm in regard to its various segments selectively for stroke survivors with moderate to severe arm paresis, or the level of performance related to different sensorimotor abilities for stroke survivors with mild arm paresis [12].

This cohort study aimed to investigate whether stroke survivors in the chronic stage of their condition (i.e., ≥6 months post-stroke) with various degrees of arm paresis, i.e., from mild to severe, could benefit from a two-week course of intensive impairment-oriented arm rehabilitation. For this purpose, the participants received daily therapy as either Arm Basis Training (moderate to severe arm paresis) or Arm Ability Training (mild arm paresis) [12], combined with individually selected technology-based arm rehabilitation, for a total of 4 h per weekday, for two consecutive weeks (ten sessions). Both standardised clinical assessments and technology-based measures were used to evaluate to what degree the patient’s motor function improved and whether other body functions (strength, spasticity/resistance to passive movement, somatosensation, or passive joint mobility) were affected in parallel, whether more use of the affected limb in the community was promoted, and whether the patient-reported emotional well-being and quality of life changed. In addition, acceptability in terms of the drop-out rate and safety, based on documented adverse events, were addressed.

## 2. Materials and Methods

### 2.1. Study Design, Setting, Data Sources and Management, and Ethical Standards

This is an observational cohort study that involved pre-testing, two weeks of intensive outpatient arm rehabilitation, and post-testing.

Two centres providing intensive impairment-oriented arm rehabilitation participated in the study, i.e., (1) the Hand and Occupational Therapy Outpatient Service Laborn, München (urban region), Germany, and (2) the Hand and Occupational Therapy Outpatient Service Laborn, Ergoldsbach (rural area), Germany. All the study interventions were performed at one of the two centres, which are equipped with the same arm rehabilitation technology and provided comparable (standardised) arm rehabilitation therapy, as described below.

All the data were collected by staff at the two participating centres, who had received training with regard to the study protocol, the assessments and therapy implemented, and the clinical report forms (CRFs) used that contained the pseudonymised data. The CRFs were digitally transmitted to and independently reviewed by the working group in Greifswald; any questions related to the data were settled through queries; database entries for the statistical analyses were performed in Greifswald and were followed by a validity check before the analysis commenced.

### 2.2. Participants

The participants in this observational study were stroke survivors with arm paresis, who intended to participate in a course of intensive arm rehabilitation at one of the two participating centres.

The eligibility criteria for the study were: age ≥ 18 years, a history of stroke (ischemic stroke, non-traumatic intracerebral haemorrhage, or subarachnoid haemorrhage; including recurrent stroke episodes) with hemiparesis, the time of the stroke being at least 6 months prior to study entry, arm paresis (Motricity Index arm score < 100 [range 0–100] [13]), available to attend a planned course of intensive arm rehabilitation for a minimum of two weeks at one of the two participating centres, and no other disease causing arm paresis (e.g., inflammatory brain disease, tumour, degenerative disease of the nervous system).

At study entry, the patients were grouped as either having mild or moderate to severe arm paresis. The criteria for mild arm paresis were: a Motricity Index arm score < 100; Motricity Index item scores for shoulder abduction, elbow flexion, and precision grip > 19; preserved selective motion capacity of the fingers; and preserved pinch grip capacity. The criteria for moderate to severe arm paresis were: a Motricity Index arm score < 100 and at least one criterion for mild arm paresis not being met.

### 2.3. Participant Characteristics

For all the participants, the following characteristics were documented at study entry: age, gender, type of stroke aetiology (i.e., ischemic stroke, non-traumatic intracerebral haemorrhage, or subarachnoid haemorrhage), side of the brain affected (i.e., left or right), number of stroke episodes in the past (i.e., one or more than one), time post-stroke (in weeks), degree of paresis (i.e., Motricity Index affected arm, Motricity Index affected leg) [13], Botulinum toxin therapy for the affected arm within 6 months prior to study entry, arm motor function of the affected arm (i.e., Box and Block Test (BBT) and Nine-Hole Peg test (NHPT) for participants with mild arm paresis or Fugl-Meyer arm motor score (FM Arm) for participants with moderate to severe arm paresis) [14,15], neuro-disability (Barthel Index) [16], emotional distress (Hospital Anxiety and Depression Scale, HADS) [17], and recruiting centre (Munich or Ergoldsbach).

### 2.4. Therapies Applied

#### 2.4.1. Intervention Description

The intervention is summarised according to the template for intervention description and replication (TIDieR) checklist [18] detailed in Table 1, and further explained in the text below.

#### 2.4.2. Therapeutic Approach Used

The therapeutic concept was to provide intensive outpatient arm rehabilitation using a training approach that was both standardised and individually adapted as needed, which was challenging and yet feasible for the individual participant, and intended to promote the recovery of motor control in the affected paretic arm.

The intended arm rehabilitation training schedule and dosage was 4 h of facility-based repetitive training on 5 days per week, for two consecutive weeks. Apart from the dosage, the training was designed to be intensive due to its repetitive structure, through an individualised adaptation that was designed to keep training tasks challenging throughout the training period (not too easy and not too difficult to complete), and close supervision and/or physical guidance (1:1 therapist–patient ratio), with the aim being to provide all the necessary information, to provide motivation in order to encourage the patients’ adherence to the training schedule, to always keep the training at the individual’s performance limit, with a clear focus on improving motor control and performance throughout the training, and to support a persistently high level of engagement by the participants.

Therapy was provided by occupational therapist staff at the two participating centres, who were experienced in arm rehabilitation post-stroke and the specific interventions applied during the study.

#### 2.4.3. Impairment-Oriented Training, IOT

Regarding the training contents, the therapy was impairment oriented [12].

For stroke survivors with moderate to severe arm paresis, the explicit therapeutic aims were: (1) to promote selective movement capacity; (1a) first, without the need to compensate for limb weight and the influence of gravity for single degrees of freedom of the arm, hand, and fingers; then (1b) as (1a), but under the affordances of weight bearing and managing the influence of gravity and external forces; and (1c) multi-joint movements under the influence of gravity and external forces. To promote these movement control capacities, stroke survivors received both the Arm Basis Training, ABT, and arm rehabilitation robot training every day, in combinations that were individually adapted.

Among the patients with mild arm paresis, who were able to handle smaller and larger objects, while still being “clumsy” during such tasks, caused by a lack of speed and precision in terms of sensorimotor control, the aim (2) was to improve their sensorimotor control across various abilities, such as the speed of finger movements, aiming, steadiness, continuous visuomotor control, finger and gross manual dexterity, as well as endurance. These stroke survivors received the Arm Ability Training, AAT, on a daily basis, again combined with technology-supported training.

The standard training schedules included either ½ h ABT plus 3 ½ h arm rehabilitation robot training, or 1 h AAT plus 3 h non-robotic technology-supported training, per day.

Both the ABT and AAT, as well as the rehabilitation technology used, were individually adapted to the participant’s level of performance/motor control by the therapist in charge.

In addition, the patients were offered patient-led training either as mirror therapy, smart glove-based game-like finger exercises, or exercises for somatosensory stimulation; the type of optional, additional patient-led training was suggested on an individual basis.

#### 2.4.4. Arm Robot Rehabilitation and Other Technology-Supported Training

The following arm rehabilitation technology was used for the participants collectively. Combinations were selected as individually indicated, i.e., most likely to support motor recovery.

##### Amadeo^®^ and Amadeo^®^/EMG

A robotic and sensor-based rehabilitation device for assistive and interactive therapies for individual finger and thumb movements; optionally used with a surface electromyography module (EMG) for neuromuscular electrical stimulation.

##### Gloreha Sinfonia

A robotic and sensor-based rehabilitation device for assistive and interactive therapies for individual finger and thumb movements.

##### Diego^®^

A robotic and sensor-based rehabilitation device for assistive and interactive therapies for the whole arm and assisted, as needed, by an “intelligent gravity compensation” (IGC) functionality.

##### Pablo^®^

A non-robotic sensor-based rehabilitation device for interactive therapies for the whole arm (fingers, hand, arm)

##### Myro

A non-robotic rehabilitation device with sensor-based surface and real objects for the training of gross motor skills and fine motor skills.

##### Rapael

A non-robotic sensor-based rehabilitation smart glove device for interactive therapies for the fingers and hand. Rapael was used for patient-led training (only).

Amadeo^®^, Diego^®^, Pablo^®^, and Myro are devices provided by the manufacturer Tyromotion (www.tyromotion.com; accessed on 1 November 2024), Rapael is provided by Neofect (www.neofect.com; accessed on 1 November 2024), and Gloreha Sinfonia is provided by Idrogenet (www.gloreha.com; accessed on 1 November 2024).

#### 2.4.5. Transfer to Daily Life and Amount of Use

Aside from the training as described above, patients were encouraged to explore and use the motor capacities of their affected arm at home as much as possible. To support the actual amount of use of the affected arm, patients received and wore an activity monitor (Armtracker ARYS™, provided by Tyromotion) during waking hours (except during on-site training hours). The activity monitor was linked to visual feedback provided by an app. Through the connected app, stroke survivors could watch their “tree of recovery” grow: every recorded arm movement caused a new leaf to sprout on their smartphone.

### 2.5. Outcome Measures

#### 2.5.1. Documentation of Training Therapy Applied

For each type of training, the time of active training (in minutes) per day was documented and, consecutively, the total training time over the course of 2 weeks (10 days of training).

The types of training for which the training time was recorded were: impairment-oriented training (as either AAT or ABT), training with Amadeo^®^, training with Amadeo^®^/EMG, training with Pablo^®^, training with Diego^®^, training with Gloreha, training with Myro, patient-led training with Rapael, patient-led somatosensory training, patient-led mirror therapy, and the individual total training time (i.e., sum across all the categories of training).

#### 2.5.2. Primary Outcome Measures

The primary outcome measures included standardised pre–post differences in the Fugl-Meyer arm motor scores (subgroup severe arm paresis) or in terms of the combined standardised pre–post differences in the NHPT and BBT scores (subgroup mild arm paresis) [14,15,19].

The standardisation of the pre–post differences was based on the standard deviation in the study sample for the Fugl-Meyer arm motor scores and the NHPT and BBT scores at the baseline. For the individually combined standardised pre–post differences in the NHPT and BBT scores, the standardised NHPT and BBT pre–post difference scores were equally weighted.

These primary outcome measures were selected because they most closely assess the motor functions intended to be promoted by the impairment-oriented training in the two subgroups, i.e., to quantify the abilities of the participants to make both isolated and coordinated joint movements and, hence, display selective motion control (Fugl-Meyer arm motor scores), or to assess the degree of the participant’s gross manual (BBT) and finger (NHPT) dexterity, respectively.

#### 2.5.3. Secondary Outcome Measures

The secondary outcome measures were the pre–post difference scores for the following measures:

- FM, passive motion/pain—Fugl-Meyer arm summary score for passive motion and pain scales (0–48);
- FM, somatosensory—Fugl-Meyer arm summary score for somatosensory sensation scale (0–12);
- Finger extension strength—maximum finger extension strength for fingers II to V simultaneously (in kg), measured using Amadeo^®^;
- Active ROM arm (in degrees)—average of the degrees of active range of motion as measured for the shoulder abduction/adduction, internal/external rotation, flexion/extension, elbow flexion/extension, forearm pro-/supination, and wrist flexion/extension, measured using Diego^®^ and Pablo^®^;
- Active ROM fingers (%)—average of the active range of motion measured as a percentage of the passive range of motion for each finger, i.e., digit I to V, and both flexion and extension, respectively, measured using Amadeo^®^;
- REPAS arm (0–32)—sum of REsistance to PASsive movement scale scores for shoulder abduction, external rotation, flexion, elbow flexion and extension, forearm supination, wrist extension, and finger extension [20];
- Finger spasticity—Modified Ashworth Scale, objectively measured for fingers II to V simultaneously, measured using Amadeo^®^;
- AOU (movements per hour)—amount of use of the affected arm in daily life (waking hours), measured with the arm tracker ARYS™ (worn on the wrist);
- HADS—Hospital Anxiety and Depression Scale (0–42) [17];
- EQ5D VAS—Visual Analogue Scale score for the EQ-5D-5L Self-Complete questionnaire—paper version [21].

#### 2.5.4. Additional Outcomes

Acceptability was assessed using the drop-out rate of the participants willing to participate, but who did not complete the two-week course of training.

The patients were asked about adverse events (AEs) on a daily basis and were documented. The distinction between AEs and serious AEs (SAEs) followed the guidance for safety reporting in clinical investigations of medical devices under the Medical Device Regulation [22].

### 2.6. Statistical Analyses

#### 2.6.1. Estimates of Therapeutic Effects

The data of all the participants with pre- and post-data on the primary outcomes were used for the analyses.

The baseline characteristics were documented using descriptive statistics, i.e., mean, standard deviation (sd), minimum and maximum, or count and relative frequency, as indicated.

The dosage of the training therapy (i.e., total training time in minutes for each type of training) was documented using descriptive statistics, i.e., mean, sd, and 95% confidence interval (95% CI).

For each outcome measure, the pre–post differences were analysed for their statistical significance using paired *t* tests. For the primary outcome measures, the *p* value for hypothesis testing was set at 0.05, for the 10 secondary outcome measures the *p* value for hypothesis testing was adjusted for multiple comparisons to 0.005.

The drop-out rates and adverse events (in categories, i.e., the type of AE and frequency categories ≤3 days, ≤6 days, and ≥7 days) were given as counts and relative frequencies.

#### 2.6.2. Exploratory Analysis of the Modifying Factors of the Therapeutic Effect

To explore the relevance of several independent variables for the magnitude of the therapeutic effect observed, a general linear model was used and multivariate analysis of variance was performed.

The primary outcome, i.e., the standardised mean difference based on the pre–post differences in the standardised Fugl-Meyer arm motor scores (subgroup severe arm paresis) or in terms of the combined standardised NHPT and BBT scores (subgroup mild arm paresis), was used as the dependent variable.

The following independent variables were used: the classification variables, namely sex, handedness, stroke type, affected side of the brain, the subgroup of arm paresis (mild or severe); and the numeric variables, namely age, number of previous stroke episodes, time post-stroke (weeks), the Motricity Index score (affected arm), total training time (minutes), and both the baseline data and pre–post change scores for the finger extension strength (kg), active ROM arm (degrees; i.e., the average of the degrees of active range of motion as measured for the shoulder abduction/adduction, internal/external rotation, flexion/extension, elbow flexion/extension, forearm pro-/supination, and wrist flexion/extension), the active ROM fingers (% of passive ROM; i.e., the average of the active range of motion, measured as a percentage of the passive range of motion for each finger, i.e., digit I to V, and both the flexion and extension, respectively), the REPAS arm score (the sum of REsistance to PASsive movement scale scores for shoulder abduction, external rotation, flexion, elbow flexion and extension, forearm supination, wrist extension, and finger extension), the HADS score (Hospital Anxiety and Depression Scale), and the EQ5D VAS score (Visual Analogue Scale score for the EQ-5D-5L Self-Complete questionnaire—paper version).

For the model, the *p* value for hypothesis testing was set at 0.05; regarding the testing of the individual independent measure’s statistical significance, the *p* value (based on type III sums of the squares) was adjusted for multiple comparisons to 0.0027 (0.05/22). No data imputation was performed, only complete datasets were used for the multivariate analysis.

### 2.7. Sample Size Calculation

The statistical corroboration of at least small within subject pre–post differences (effect size d = 0.2) with a pre-defined alpha error probability of 0.05 and a power (1—beta error probability) of 0.80, required a sample of 52 participants [23]. To support the subgroup analyses, i.e., separate analyses for the participants with either mild or moderate to severe arm paresis, a study sample of 100 participants was pre-planned and recruited.

### 2.8. Bias

Since the staff at the two participating centres could not be blinded regarding the intervention, there is a risk of information bias. The standardised clinical assessments used were amended using objective technical measurements not influenced by human expectations and, hence, secondary measures with little or no risk of information bias were integrated.

The potential risk of attrition bias was dealt with by close, 1:1 therapeutic supervision, guidance, and adaptation of the therapy during the intervention period, ensuring a high rate of acceptability and fidelity.

## 3. Results

One hundred chronic stroke survivors were recruited to participate in the observational study, while receiving their arm rehabilitation.

Ninety-seven participants completed a two-week course of training and both pre- and post-test assessments; three participants did not complete the course. Accordingly, the drop-out rate was 3% (3/100).

The drop-outs occurred after 1 (#1), 5 (#2), and 9 days (#3) of training. The reasons were discomfort with the study procedures, i.e., the assessment of emotional well-being and wearing an activity monitor outside therapy (#1), related to the coronavirus pandemic (#2), and a lack of motivation (observed throughout the training) (#3).

The data on the ninety-seven chronic stroke survivors receiving arm rehabilitation and completing the study procedures were used for further analyses. Their baseline characteristics are presented in Table 2.

The study population included participants with a considerable age distribution, both genders, and different types of stroke aetiology. The majority had moderate to severe arm paresis (93%), a smaller subgroup had mild arm paresis (7%). In addition, considerable variability in the time post-stroke, ranging from 6 months to many years, the degree of arm and leg paresis (Motricity Index), the arm motor function of the affected arm (BBT/NHPT; or, FM Arm), neuro-disability (Barthel Index), and emotional distress (HADS) was documented among the participants.

The type and dosage of the training applied over the two-week course of training are detailed in Table 3.

On average, the participants received about forty hours of training, i.e., as the intended 4 h per training day. Of that time, 5.5 h (or slightly more than 0.5 h per day) was IOT as either ABT or AAT. The different forms of technology-based arm rehabilitation, administered in regard to a 1:1 therapist–patient ratio, were all applied and collectively covered much of the reminder of the training time, i.e., approximately 33 h in total, on average (198 min per participant and per day). Optionally offered additional patient-led forms of training were used to a limited extent only, i.e., approximately 2 h in total, on average (12 min per participant and per day).

The outcomes of the two-week course of therapy are presented in Table 4.

The primary outcome measures indicated an improvement in the motor function of the patients of a moderate size (standardised mean difference, SMD 0.42, 95% CI 0.36–0.49; n = 97). At the same time, the arm and hand spasticity/resistance to passive movement (averaged across eight different passive joint movements, REPAS) and finger spasticity improved (n = 97). Both the active range of arm movements (averaged across different joints and reciprocal movement directions; mean 16 degrees, 95% CI 13.6–18.4; n = 76) and finger movements (averaged across digits I to V and reciprocal movement directions; mean 4.9% of passive ROM, 95% CI 2.9–6.9; n = 92) improved, while the finger strength did not (n = 96). The passive joint mobility of the affected arm and associated pain was slightly improved, as was the somatosensory sensation among the participants with moderate to severe arm paresis (n = 90). The amount of use of the affected arm (outside the therapeutic facility) improved (AOU; n = 81), as did the participants’ emotional distress (HADS; n = 92) and quality of life (EQ5D VAS; n = 93).

The type and frequency of occurrence of adverse events (AEs) are detailed in Table 5.

No AEs qualified as severe (SAEs) [20]. Most AEs were training related. They either did not require any intervention, could be dealt with by the therapist in charge during the training (e.g., by adding in short breaks or adjusting the training tasks), or only required a period of rest at home after training (e.g., for 0.5 to 1 h). About half of the participants (51%) experienced an AE with a bimodal distribution, i.e., many participants experienced AEs during a few days (mostly the first few days) of training only (24/49, 49%), while for a third of the participants, AEs accompanied the majority if not all of the training days (17/49, 35%). Tiredness during or after training was the most frequently documented AE. This complaint was reported by a quarter of participants (25/97, 26%), either during a few days of training only (13/25, 52%) or more frequently (12/25, 48%). Shoulder discomfort/pain was the second most frequently noted AE (10/97, 10%), with the majority of those affected reporting this AE during a few (the first) days of training (6/10, 60%) only. The third most common AE was muscle discomfort, which was reported by 6% of the study population (6/97, 6%).

The results of the multivariate exploratory analysis of the modifying factors in terms of the therapeutic effects observed (primary outcome) are detailed in Table 6.

The multivariate exploratory analysis involved the use of a general linear model that was based on 70 complete datasets and indicated a significant effect modification (F(23,69) = 2.10; *p* = 0.0161). From the 22 independent variables analysed, only the variable active ROM arm (degrees; i.e., the average of the degrees of active range of motion as measured for the shoulder abduction/adduction, internal/external rotation, flexion/extension, elbow flexion/extension, forearm pro-/supination, and wrist flexion/extension) was statistically corroborated as a significant modifier of the therapeutic effect (F(1,69) = 14.38, *p* = 0.0004); the active ROM of the arm at the baseline was associated with bigger improvements at the post-test stage. A follow-up univariate model using data from all the participants in terms of that measure (active ROM Arm), revealed a small explanatory strength in the measure, accounting for 10% of the variance in the primary outcome measure only (n = 81; R^2^ = 0.1002). The subgroup of arm paresis (mild or severe) (F(1,69) = 7.86, *p* = 0.0074) did not reach the pre-defined threshold of statistical significance (i.e., *p* = 0.0027). The effect size for the primary outcome in the two respective subgroups was on average somewhat higher in the subgroup with severe arm paresis (severe arm paresis: SMD 0.43, 95% CI 0.37–0.50, n = 90; mild arm paresis: SMD 0.33, 95% CI 0.05–0.60, n = 7).

## 4. Discussion

While there is an enhanced period of neuroplasticity to support functional recovery during the first few months after stroke [8] and, hence, a reduction in disability [24], we have rather limited knowledge on whether and how functional recovery can still be promoted in the chronic phase after stroke [25,26].

Here, we report on a cohort study, where stroke survivors in the chronic phase (i.e., ≥6 months post-stroke, on average >2 years) received intensified impairment-oriented outpatient arm rehabilitation treatment in an urban (Munich) or a rural (Ergoldsbach) occupational therapy centre in Germany. On 10 consecutive weekdays over a course of 2 weeks, they received 4 h of daily arm rehabilitation training. Therapy was provided in a 1:1 therapist–patient ratio setting.

All the patients received daily impairment-oriented training as either Arm Basis Training (moderate to severe arm paresis; n = 90) or Arm Ability Training (mild arm paresis; n = 7), in addition to technology-supported training, with individual combinations selected from six types of training/technologies.

Through the use of a high dosage of both standardised and individualised impairment-oriented arm motor rehabilitation training, the focus on training at the individual’s performance limit, the encouragement for patients to display engaged training behaviour, the aim to continuously improve the individual’s motor control capabilities of the affected arm, and the explicit promotion of the transfer of any regained motor capacity to everyday life, the training can be regarded as “intensive”. With a drop-out rate of only 3%, the training protocol was nevertheless very acceptable to the participants.

With this training set-up, two weeks of intensive arm rehabilitation training improved the participants’ motor function. The effect size (SMD 0.42, 95% CI 0.36–0.49) can be considered moderate [27] and, consequently, clinically relevant (compared to Table 4). The estimate was fairly precise (had a relatively small confidence interval) pointing to a low degree of heterogeneity in terms of the therapeutic effect in the study population, even though the participants did show variation with regard to their baseline characteristics (compared to Table 2). Hence, the estimated effect can be considered representative of the recruited clientele and the therapeutic approach applied. As the primary outcomes were focused on addressing arm and hand functions, the results indicate that motor recovery was still achievable for stroke survivors in the chronic stage when the therapy involved training that explicitly, specifically, intensively, and comprehensively addressed the motor control to be regained in a structured, standardised manner, with individual adaptations and close therapeutic supervision.

Since the vast majority of the participants had moderate to severe arm paresis (e.g., 92.8%), the observed magnitude of the therapeutic effect is mainly representative of this clientele.

For the Fugl-Meyer arm motor score, this equates to a gain of 4 points and was, hence, borderline when it comes to the minimal clinically important difference (MCID) among chronic stroke survivors (MCID 4.25 to 7.25 points [28]). Previous systematic review data on the effects of electromechanical and robot-assisted arm training reported a somewhat lower effect size (SMD 0.32, 95% CI 0.18–0.46, 41 studies, 1452 participants), even though the systematic review included many stroke survivors in the subacute phase, typically showing higher improvement rates [29]. Compared to other studies with high-intensity training for persistent upper limb dysfunction in the chronic phase post-stroke that documented on average a gain in the Fugl-Meyer arm motor scores of 6 points after 90 h of training [25], or 9 points after 300 h of therapy [26], the dosage applied in this study was considerably lower (i.e., 40 h) and, hence, the gains achieved appear somewhat higher in relation to the dosage applied.

The Fugl-Meyer arm motor score quantifies the abilities of the participants to make both isolated and coordinated joint movements. Improved motor function as assessed using such score can be related to either improved strength or reduced intrusions related to synergies [30].

In the present study, the beneficial effect on arm motor function was demonstrated not only due to the use of clinical assessment instruments, but also through the use of comprehensive technology-based objective measures of active range of motion (aROM) capabilities. Indeed, a substantial improvement in aROM capabilities, as assessed for many joints in the arm and the fingers, was documented (compared to Table 4). The fact that the mean improvement rates for various joints (in both the paretic arm and fingers) were individually analysed and, overall, indicated a training-induced improved motion capacity, points to a substantial and widespread therapeutic effect on the selective motion capacity in regard to individual degrees of freedom for arm and finger joints. This favours the notion that the therapeutic approach induced (partial) restoration (and not merely compensation) of the motor function among stroke survivors in the chronic phase of their condition.

As longitudinal studies have shown that for most stroke patients spontaneous neurological recovery and behavioural restitution plateaus within the first 10 weeks post-stroke, it has been assumed that any further functional improvement later on is likely to reflect compensation, i.e., performing movements or tasks with atypical movement patterns at the expense of movement quality, or even by performing tasks with a different limb [31]. Here, however, it was demonstrated that more than 6 months post-stroke selective movement control for individual joints could be further improved when an intensive impairment-oriented training was applied that, at the individual level of motor control, systematically and repetitively trained selective movement control, with the goal of gradually improving this body function.

The exploratory multivariate analysis of a total of 22 variables revealed that the observed therapeutic effects were largely independent of the other patient characteristics assessed, such as age, sex, type of stroke, or time after stroke (≥6 months post-stroke), handedness, degree of spasticity, emotional distress (level of anxiety or depressive symptoms when commencing the training period), or self-reported quality of life, and where applicable, their change over time (from pre- to post-test). The only variable that could be substantiated as a modifier was the active range of motion in the arm. A better active range of motion control at the baseline was associated with somewhat higher gains (primary outcome) from training, explaining approximately 10% of the inter-individual variance in terms of such gains. The fact that the magnitude of the improvement in the primary outcome was not significantly linked to parallel changes in other outcomes, like spasticity or emotional distress, is also noteworthy and supports the notion that the observed improvements in active motor control (primary outcome) were not merely a reflection of the mediation by other clinically relevant aspects that (on average) showed improvements during the study period.

This adds to our knowledge for the benefit of clinical decision-making. The observed therapeutic effectiveness of the type of training offered applies to those who were eligible to participate (and not only to a subgroup of them or being largely dependent on some of their characteristics). The approach can, therefore, be considered externally valid for stroke survivors with these characteristics.

The stroke survivors seeking arm rehabilitation services at the two participating centres, one in an urban and one in a rural area, had diverse baseline characteristics as might be considered typical for a rather non-selected utilisation clientele (compared to Table 2). In this context, it is important to note that despite this diversity, the therapeutic gain achieved was fairly independent from such differences and, hence, is more generally applicable.

Further observed therapeutic effects are noteworthy (compared to Table 4).

The improvements in the motor function were complemented by a relevant improvement in the spasticity/resistance to passive movement (i.e., 10% improvement in the REPAS arm spasticity score) and slight improvements in the passive mobility and somatosensation in the paretic limb.

The amount of use of the affected arm at home and in the community improved equally, from pre- to post-testing (on average by five movements per hours, relating to a 13% improvement over the baseline; the baseline scores were on average 37.7 movements per hour), indicating a transfer of the regained motor capacities and use of motor behaviour from the training situation to the community.

While the magnitude of the effect on the patients’ quality of life (EQ-5D VAS mean difference 5.5) was below what can be considered the MCID (EQ-5D, 8.6 [32]), the observed improvement rates (HADS mean difference −1.7) met the MCID criterion for emotional distress (HADS 1.7 [33]).

The adverse event (AE) statistics (compared to Table 5) showed that AEs were experienced by about a half of the participants and, frequently, only during the first few days (mostly at the beginning of training) of training. No serious adverse events were noted and all AEs could be handled by simple means, including training adjustments or rest after training. The high intensity of the training caused tiredness in a quarter of the participants. The next most frequently noted AE was shoulder discomfort or pain. The type, frequency, and severity of the AEs were well within the expected spectrum for an intensive arm motor training involving stroke survivors. The data help to provide information about the expected unwanted effects on future trainees and point to the necessity of therapeutic supervision.

Overall, it is noteworthy that the structured high-intensity impairment-oriented training schedule (e.g., 4 h daily motor practice at the individual’s motor control/performance limit for a few weeks), with close therapeutic supervision, promoted improved motor control with a transfer of gained motor capacities to the actual amount of use among chronic stroke survivors, even those with severe arm paresis.

With regard to the implementation of such an approach, two aspects reflecting the resources used are worth mentioning. Its implementation as a centre-based outpatient therapy provided the necessary human and technology resources for its conduct. Firstly, additional cost-effectiveness analyses that compare the achieved clinical benefits of the therapy administered to the costs incurred for the resources used [34] could provide stroke survivors, clinicians, and healthcare administrators with additional guidance for their therapeutic and resource allocation decisions. Secondly, while it has been shown that training at home with remote supervision (i.e., tele-rehabilitation) had similar effects when the training performed was comparable [35], future implementation of the therapeutic approach investigated as training carried out at with remote supervision would, for many of our participants, not be feasible. The majority of participants in this study had severe arm paresis and, hence, needed physical support (either from a therapist or robot technology) to assist them with the repetitive training. With this fact in mind, the resources to be made available at home would not favour its implementation as home-based training.

The limitations of the study are related to its methodology. Without a control group receiving training with different content and/or dosage, the data reported apply to this complex approach without the possibility to learn about the relevance of the individual components, e.g., whether the selected training tasks, their repetition rates, or the type of feedback provided were the most important in regard to the improvements induced [36]. Based on our knowledge, gained from a previous multicentre RCT, it is likely that a combination of the comprehensive range of specifically selected training tasks and their application in intensive, repetitive, and engaged training at the individual’s performance limit, with a sufficient dosage, are the most important aspects [19]. While the use of technology-based objective measurements of motor function assured observer-independent outcome measurements and supports the notion of the restitution of function (as opposed to improved compensatory behaviour), the clinical assessment was not blinded for the intervention and, therefore, there is a risk of information bias. While the type of measures used were adequate for the research questions addressed, the use of additional tests could have resulted in a more complete picture of the therapeutic effects achieved. In regard to the clinical side, the addition of an arm capacity test, like the Action Research Arm Test, could have indicated any transfer of improved motor control to capacities in regard to arm and hand activities and dexterity [37]. Technically, a more refined kinematic approach could have provided more detailed information to differentiate compensatory strategies from motor recovery after stroke. While kinematic variables do not have higher levels of responsiveness over objective clinical outcome measures in adults with chronic stroke, they have the advantage of characterising the quality and structure of the movement and, hence, can support a better understanding of the underlying neural mechanisms of functional improvements [30,38]. Equally, with the use of brain activity analyses (e.g., with multi-channel EEG), insights about the neural mechanisms related motor control improvement could be gained [39]. Furthermore, we do not know to what extent a longer course of training would have generated greater improvements and, as no systematic follow-up was conducted, the long-term effects remain unknown. In addition, subgroup analyses for stroke survivors with moderate to severe versus mild arm paresis were not feasible, as the vast majority of the study participants had moderate to severe arm paresis. And, in spite of the comprehensive assessment of the treatment effects on various aspects relevant for arm rehabilitation in this study, any potential transfer to other motor domains (e.g., leg motor control and mobility) was not addressed as a research question [36].

## 5. Conclusions

Overall, this study adds to our knowledge that in the chronic stage of stroke, including among those with severely paretic arms, a two-week course of intensive impairment-oriented and technology-supported arm rehabilitation is very acceptable for stroke survivors and can significantly improve motor function through (partial) the restoration of function, with a transfer effect in regard to such use in everyday life of the affected limb. Adverse events were not infrequent, yet not severe, and were manageable within the therapeutic setting. Accordingly, a structured high-intensity impairment-oriented training schedule (e.g., 4 h daily motor practice at the individual’s motor control/performance limit for a few weeks), with close therapeutic supervision, might be a way to promote arm motor function after stroke in the chronic stage, even among stroke survivors with severe arm paresis. The data supports the notion that such a therapeutic approach can induce further recovery of motor control, even in the chronic phase after stroke. Exploratory analysis of the modifiers indicated that the magnitude of the therapeutic effect was largely independent of individual participant characteristics and applied similarly to all the stroke survivors enrolled.

## Figures and Tables

**Table 1:** Intervention description and replication (TIDieR) checklist [18].

Item	Description
Name	Impairment-oriented and technology-supported rehabilitation for arm paresis in chronic stroke patients.
Why	The study followed the rationale (and hypothesis) that motor recovery is still achievable for stroke survivors at the chronic stage, when therapy offers training that explicitly, specifically, intensively, and comprehensively addresses the type of motor control to be regained, i.e., the ability to move the arm and hand according to its various segments selectively in the case of moderate to severe arm paresis, or a higher level of performance in relation to sensorimotor abilities among stroke survivors with mild arm paresis.
What	Impairment-oriented training (as either AAT or ABT), training with Amadeo^®^, training with Amadeo^®^/EMG, training with Pablo^®^, training with Diego^®^, training with Gloreha, training with Myro, patient-led training with Rapael, patient-led somatosensory training, and patient-led mirror therapy.
Who provided	Occupational therapists at the two participating centres, who were experienced in arm rehabilitation post-stroke and the specific interventions applied.
How	Therapy was provided individually, in a 1:1 patient–therapist setting.
Where	All the study interventions were performed at one of the two participating occupational therapy centres, which are equipped with the same arm rehabilitation technology (as described above).
When and how much	The arm rehabilitation training schedule and dosage was 4 h of facility-based repetitive training, 5 days per week, for two consecutive weeks. The standard training schedule included either ½ hour ABT plus 3 ½ h arm rehabilitation robot training (moderate to severe arm paresis), or 1 h AAT plus 3 h non-robotic technology-supported training per day (mild arm paresis). In addition, the patients were offered patient-led training.
Tailoring	The training was designed to be intensive due to its repetitive structure and as a result of individualised adaptations that were intended to keep training tasks challenging throughout the training period (not too easy and not too difficult to complete). For this purpose, both the ABT and AAT, as well as the rehabilitation technology used, were individually adapted to the participant’s level of performance/motor control by the therapist in charge.
Modifications	According to the training standards described above, therapists adjusted the intervention to the individual’s current level of motor control capabilities throughout the course of treatment.
How well	Close supervision and/or physical guidance (1:1 therapist–patient ratio) was provided to participants with the aim being to provide all the necessary information, to provide motivation in order to encourage the patients’ adherence to the training schedule, to always keep the training at the individual’s performance limit, with a clear focus on constantly improving motor control capabilities throughout the training, and to support a persistently high level of engagement by participants. In addition, the transfer of regained motor capacities to everyday life was addressed and encouraged, including smartphone-based feedback on the motor activity/actual use of the paretic arm in the community. The training time for the individual components of the therapy were continuously monitored, as was the patients’ arm activity in the community.

Abbreviations: AAT—Arm Ability Training; ABT—Arm Basis Training.

**Table 2:** Study population characteristics (n = 97).

	Mean/Sd	Min–Max	n
	n (%)	n (%)	n (%)
**Age** (mean/sd, min–max)	54.8/13.6	19–80	
**Sex** (female, male) (n (%))	35 (36%)	62 (64%)	
**Handedness** (right, left) (n (%))	92 (95%)	5 (5%)	
**Stroke type** (ischemic, ICH, SAB) (n (%))	59 (61%)	35 (36%)	3 (3%)
**Affected side of the brain** (left, right) (n (%))	34 (35%)	63 (65%)	
**Stroke episodes** (one, more than 1) (n (%))	85 (88%)	12 (12%)	
**Time post-stroke** (weeks) (mean/sd, min–max)	111/106	26–721	
**Subgroup arm paresis** (mild ^b^, severe ^a^) (n (%))	7 (7%)	90 (93%)	
**MI, affected arm** (0–100) (mean/sd, min–max)	43/18	15–100	
**MI, affected leg** (0–100) (mean/sd, min–max)	56/19	19–100	
**Botulinum toxin N** (within 6 months) (n (%))	12 (12%)		
**Barthel Index** (0–100) (mean/sd, min–max)	82/15	25–100	
**HADS** (0–42) (mean/sd, min–max)	12.6/7.9	1–32	93 ^1^
**FM Arm** ^a^ (0–66) (mean/sd, min–max; n)	17.4/9.7	4–47	90
**BBT** ^b^ (blocks/minute) (mean/sd, min–max; n)	13.9/12.3	6–41	7
**NHPT** ^b^ (s) (mean/sd, min–max; n)	136/81.7	51–225	7
**Recruiting centre** (Munich, Ergoldsbach)	73	24	

Abbreviations: BBT—Box and Block Test; FM Arm—Fugl-Meyer arm motor score; HADS—Hospital Anxiety and Depression Scale; ICH—intracerebral haemorrhage; MI—Motricity Index; NHPT—Nine-Hole Peg Test; SAH—subarachnoid haemorrhage; min—minimum; max—maximum; sd—standard deviation. Superscript letters (mild ^b^, severe ^a^) indicate the different subgroups and the tests used for the baseline assessment, respectively. Note, ^1^ four participants did not want to disclose their personal emotional information. To promote readability variable names are provided bold.

**Table 3:** Type and dosage of training therapies (n = 97).

Type of Training	Training Time (Minutes)	
	Mean/Sd	[95% CI]
**Impairment-oriented training**	335.3/96.4	[315.8–354.7]
**Training with Amadeo^®^**	705.8/222.7	[660.9–750.7]
**Training with Amadeo^®^/EMG**	193.0/162.0	[160.3–225.6]
**Training with Pablo^®^**	398.7/148.5	[368.7–428.6]
**Training with Diego^®^**	425.6/173.5	[390.6–460.5]
**Training with Gloreha**	163.9/154.0	[132.9–194.9]
**Training with Myro**	94.9/152.4	[64.2–125.7]
**Patient-led training with Rapael**	0.15/1.5	[−0.2–0.5]
**Patient-led somatosensory training**	90.9/130.6	[64.5–117.2]
**Patient-led mirror therapy**	28.2/86.3	[10.9–45.6]
**Total training time**	2437.6/517.6	[2333.3–2541.9]

Note: The data provided are the time of active training (in minutes) per type of training (and total training time) over the course of 2 weeks (10 days of training). All participants received impairment-oriented training as either Arm Basis Training (severe arm paresis, n = 90) or Arm Ability Training (mild arm paresis, n = 7). Amadeo^®^, Pablo^®^, Diego^®^, and Myro are upper extremity rehabilitation devices provided by Tyromotion GmbH; Amadeo/EMG—Amadeo combined with the surface electromyography module (EMG) and neuromuscular electrical stimulation (NMES). Gloreha is an upper extremity rehabilitation device provided by Idrogenet srl. Rapael is an upper extremity rehabilitation device provided by Neofect.

**Table 4:** Pre–post differences for primary and secondary outcome measures (n = 97).

	n	Mean [95% CI]	*p* (t)
**Primary outcome measures**			
**SMD** (FM Arm or NHPT/BBT)	97	0.42 [0.36–0.49]	<0.0001 *
**Secondary outcome measures**			
**FM, passive motion/pain** (0–48)	90	2.1 [1.4–2.8]	<0.0001 *
**FM, somatosensory** (0–12)	90	0.5 [0.2–0.8]	0.0026 *
**Finger extension strength** (kg)	96	0.03 [−0.01–0.07]	0.1343
**Active ROM arm** (degrees)	76	16.0 [13.6–18.4]	<0.0001 *
**Active ROM fingers** (%)	92	4.9 [2.9–6.9]	<0.0001 *
**REPAS arm** (0–32)	97	−3.4 [−4.0–−2.9]	<0.0001 *
**Finger spasticity** (MAS scores)	97	−0.6 [−0.9–−0.3]	<0.0001 *
**AOU** (movements per hour)	81	5.0 [1.7–8.3]	0.0039 *
**HADS** (0–42)	92	−1.7 [−2.6–−0.8]	0.0002 *
**EQ5D VAS** (0–100)	93	5.5 [2.2–8.8]	0.0012 *

Abbreviations and explanations: 95% CI—95% confidence interval; active ROM arm—the average of the degrees of active range of motion as measured for the shoulder abduction/adduction, internal/external rotation, flexion/extension, elbow flexion/extension, forearm pro-/supination, and wrist flexion/extension; active ROM fingers—the average of the active range of motion, measured as the percentage of passive range of motion for each finger, i.e., digit I to V, and both flexion and extension, respectively; AOU—amount of use of the affected arm in daily life (waking hours), as measured with the arm tracker ARYS™ (worn on the wrist); BBT—Box and Block Test; EQ5D VAS—Visual Analogue Scale score for the EQ-5D-5L Self-Complete questionnaire—paper version; finger spasticity—Modified Ashworth Scale as objectively measured for fingers II to V simultaneously; FM Arm—Fugl-Meyer arm motor score; FM, passive motion/pain—Fugl-Meyer arm summary score for passive motion and pain scales; FM, somatosensory—Fugl-Meyer arm summary score for somatosensory sensation scale; HADS—Hospital Anxiety and Depression Scale; ICH—intracerebral haemorrhage; MI—Motricity Index; n—number of participants with both pre- and post-test data for the respective assessment; NHPT—Nine-Hole Peg Test; min—minimum; max—maximum; *p* (t)—*p* value for paired *t* test; REPAS arm—sum of REsistance to PASsive movement scale scores for shoulder abduction, external rotation, flexion, elbow flexion and extension, forearm supination, wrist extension, and finger extension; SMD—standardised mean difference based on pre–post differences in the standardised Fugl-Meyer arm motor scores (subgroup severe arm paresis) or in terms of the combined standardised NHPT and BBT scores (subgroup mild arm paresis). Note: For the primary outcome measures, the *p* value for hypothesis testing was set at 0.05, and for the 10 secondary outcome measures, the *p* value for hypothesis testing was adjusted for multiple comparisons to 0.005. * Denotes a *p* value below the threshold for statistical significance.

**Table 5:** Type and frequency of adverse events (AEs) (counts) (n = 97).

Type of AE	Frequency of Occurrence			
	NumberAffected	≤3 Days	≤6 Days	≥7 Days
**Any adverse event**	49	24	8	17
**Training perceived as strenuous**	3	1	0	2
**Tiredness (during/after training)**	25	13	3	9
**Tiredness (lack of sleep) ^u^**	2	2	0	0
**Nervousness**	2	2	0	0
**Emotional distress ^u^**	1	1	0	0
**Headaches**	3	2	0	1
**Neck discomfort/pain**	3	2	1	0
**Shoulder discomfort/pain**	10	6	2	2
**Shoulder discomfort/pain** (non-paretic) **^u^**	1	1	0	0
**Finger discomfort/pain**	2	2	0	0
**Lumbar discomfort/pain**	1	1	0	0
**Leg discomfort/pain**	3	2	0	1
**Muscle discomfort**	6	5	1	0
**Neuropathic pain**	2	1	1	0
**Increased muscle tension perception**	4	4	0	0
**Falls (during training)**	1	1	0	0
**Falls (in the community) ^u^**	2	2	0	0
**Epileptic seizure ^u^**	2	2	0	0
**Dizziness** (cardiovascular)** ^u^**	2	2	0	0
**Conjunctivitis ^u^**	1	1	0	0

Abbreviations and explanations: Each episode of an AE was documented using free-text entries in clinical report forms and was dated (participants could have more than one AE on a given day of training). All the AEs were classified as mild and could be managed either without intervention, by adjusting the training, including breaks, oral fluid intake, or a period of rest after training; one epileptic seizure needed medical attention and a change of medication; AEs were considered to be related to training unless indicated otherwise; u—AE considered unrelated to training.

**Table 6:** Multivariate analysis of modifying factors in terms of the therapeutic effects observed (primary outcome) (n = 70).

Independent Variable	d.f.	F Value	*p*
**Stroke type** (ischemic, ICH, SAB)	2	0.68	0.5100
**Stroke episodes** (one, more than one)	1	0.45	0.5035
**Time post-stroke** (weeks)	1	1.20	0.2783
**Subgroup of arm paresis** (mild or severe)	1	7.86	0.0074
**Age** (years)	1	0.02	0.8767
**Sex** (female, male)	1	0.01	0.9323
**Handedness** (right, left)	1	0.15	0.6974
**Affected side of the brain** (right, left)	1	0.00	0.9588
**MI, affected arm** (0–100)	1	0.41	0.5263
**Finger extension strength** (kg)**, pre-test**	1	0.17	0.6836
**Finger extension strength** (kg)**, score change**	1	5.39	0.0248
**Active ROM arm** (degrees)**, pre-test**	1	14.38	0.0004 *
**Active ROM arm** (degrees)**, score change**	1	0.03	0.8569
**Active ROM fingers** (% of passive ROM)**, pre-test**	1	0.38	0.5428
**Active ROM fingers** (% of pROM)**, score change**	1	0.54	0.4650
**REPAS arm score, pre-test**	1	0.31	0.5818
**REPAS arm score, score change**	1	0.33	0.5707
**HADS, pre-test**	1	0.97	0.3311
**HADS, score change**	1	0.81	0.3739
**EQ5D VAS, pre-test**	1	0.49	0.4880
**EQ5D VAS, score change**	1	2.33	0.1337
**Total training time** (minutes)	1	0.03	0.8681

Abbreviations and explanations: d.f.—degrees of freedom; active ROM arm (degrees)—the average of the degrees of active range of motion as measured for the shoulder abduction/adduction, internal/external rotation, flexion/extension, elbow flexion/extension, forearm pro-/supination, and wrist flexion/extension); active ROM fingers (% of passive ROM)—the average of the active range of motion, measured as a percentage of the passive range of motion for each finger, i.e., digit I to V, and both flexion and extension, respectively; EQ5D VAS—Visual Analogue Scale score for the EQ-5D-5L Self-Complete questionnaire, paper version; HADS—Hospital Anxiety and Depression Scale; ICH—intracerebral haemorrhage; MI—Motricity Index; REPAS arm score: the sum of REsistance to PASsive movement scale scores for shoulder abduction, external rotation, flexion, elbow flexion and extension, forearm supination, wrist extension, and finger extension; SAH—subarachnoid haemorrhage; score changes: the difference between the pretest and post-test scores. The primary outcome, i.e., the standardised mean difference based on the pre–post differences in the standardised Fugl-Meyer arm motor scores (subgroup severe arm paresis) or in terms of the combined standardised NHPT and BBT scores (subgroup mild arm paresis), was used as the dependent variable. Provided in the table are the F statistics for the potential modifiers (independent variables tested simultaneously). For the model, the *p* value for hypothesis testing was set at 0.05; regarding the testing of the individual independent measure’s statistical significance the *p* value (based on type III sums of the squares; the respective F values are presented) was adjusted for multiple comparisons to 0.0027 (0.05/22). No data imputation was performed; only complete datasets were used for the multivariate analysis, resulting in 70 datasets for the analysis. * Denotes a *p* value below the threshold for statistical significance.

## Data Availability

The data can only be used for the purpose of the study and in regard to those participants that had agreed by providing written informed consent.

## Clinical Trial Register

The observational study was registered with the German Clinical Trial Register (identifier: DRKS00032194).
